# Nanobiotechnology approaches for cardiovascular diseases: site-specific targeting of drugs and nanoparticles for atherothrombosis

**DOI:** 10.1186/s12951-022-01279-y

**Published:** 2022-02-08

**Authors:** Haikun Liu, Geoffrey Pietersz, Karlheinz Peter, Xiaowei Wang

**Affiliations:** 1 Molecular Imaging and Theranostics Laboratory, Baker Heart and Diabetes Institute, 75 Commercial Road, Melbourne, VIC 3004 Australia; 2 Atherothrombosis and Vascular Biology Laboratory, Baker Heart and Diabetes Institute, Melbourne, VIC Australia; 3 Burnet Institute, Melbourne, VIC Australia; 4 Department of Cardiometabolic Health, University of Melbourne, VIC, Australia; 5 Department of Medicine, Monash University, Melbourne, VIC Australia; 6 La Trobe Institute for Molecular Science, La Trobe University, Melbourne, VIC Australia

**Keywords:** Antibodies, Atherosclerosis, Gene delivery, Nanoparticles, Targeted drug delivery, Thrombosis

## Abstract

Atherosclerosis and atherothrombosis, the major contributors to cardiovascular diseases (CVDs), represent the leading cause of death worldwide. Current pharmacological therapies have been associated with side effects or are insufficient at halting atherosclerotic progression effectively. Pioneering work harnessing the passive diffusion or endocytosis properties of nanoparticles and advanced biotechnologies in creating recombinant proteins for site-specific delivery have been utilized to overcome these limitations. Since CVDs are complex diseases, the most challenging aspect of developing site-specific therapies is the identification of an individual and unique antigenic epitope that is only expressed in lesions or diseased areas. This review focuses on the pathological mechanism of atherothrombosis and discusses the unique targets that are important during disease progression. We review recent advances in site-specific therapy using novel targeted drug-delivery and nanoparticle-carrier systems. Furthermore, we explore the limitations and future perspectives of site-specific therapy for CVDs.

**Graphical Abstract:**

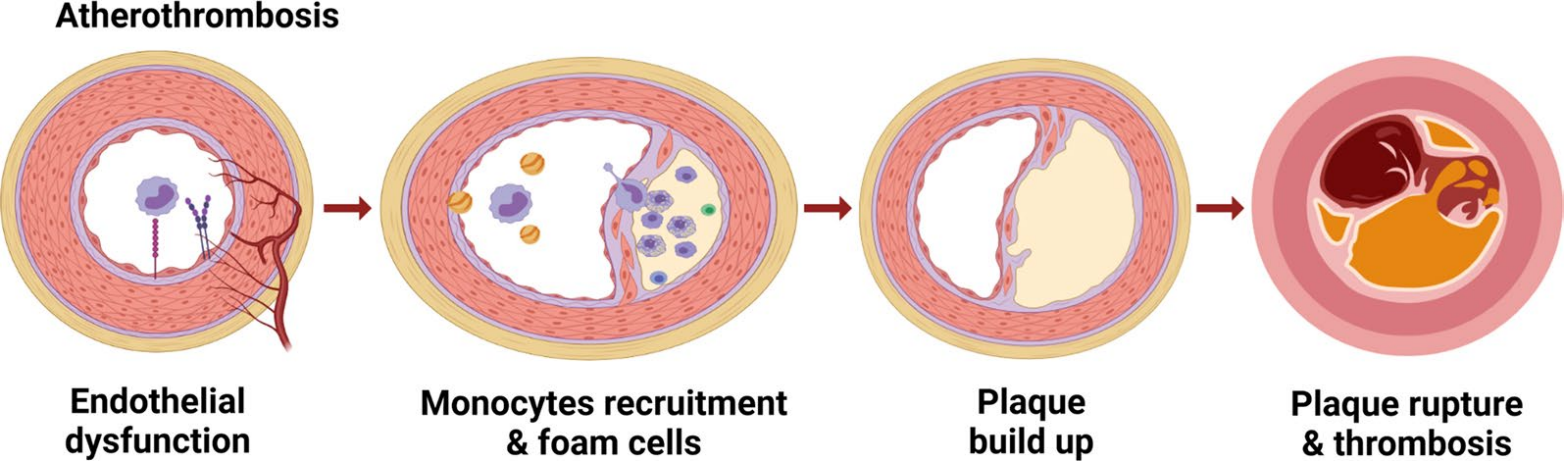

## Introduction

Cardiovascular diseases (CVD) are the leading cause of death globally, resulting in more than 17 million deaths per year [1]. The primary causes of the global CVD burden are ischemic heart disease and stroke (49% and 35% of the total burden, respectively) [2]. The main contributor to ischemic heart disease is atherosclerosis and its rupture, a process known as atherothrombosis [3]. Atherosclerosis of the major intracranial and carotid arteries also results in vessel alterations, ranging from minor wall thickening to hemodynamically substantial luminal stenosis, and is the leading cause of ischemic stroke globally [4]. Therefore, the prevention and treatment of atherosclerosis and atherothrombosis are essential to relieve the burdens of CVDs worldwide.

Atherosclerosis is a chronic inflammatory disease accompanied by lipid deposition, smooth muscle cell (SMC) proliferation, and plaque formation [5]. Unhealthy lifestyles such as poor diet quality, sedentariness, exposure to air pollution and noise, sleep deprivation, and psychosocial stress increase the risk of atherosclerosis [6]. The traditional applications of drugs are limited by insufficient effectiveness, poor distribution, and lack of selectivity [7].

Current pharmacological treatments focus on controlling the risk factors for atherosclerosis, such as glucose-lowering, lipid-lowering, and anti-hypertensive drugs. However, the efficacy of these drugs in ameliorating or reversing atherosclerotic events has been limited and further studies are needed. The most effective medications for the prevention of MI and ischemic stroke are anti-platelet and anti-coagulation therapies, which have also been associated with bleeding complications [8]. Therefore, many groups have researched either targeted drug delivery or controlled carrier systems to overcome these limitations. Drawing on the success of targeted drug delivery in the cancer field, antibody-based therapy has been very successful over the last 15 years [9]. Similarly, CVD researchers have recently harnessed the targeting ability of antibodies for the treatment of atherosclerosis [10].

Using ligands that target specific biomarkers which are expressed and upregulated during the process of atherothrombosis allows for direct regulation of inflammation and delivery of drugs to specific disease locations. Another widely researched area for CVD therapy is nanoparticles (NPs) for the delivery of therapeutic compounds, due to their optimized physiochemical and biological properties [11]. NPs can be synthesized from organic and inorganic materials, including lipids, proteins, synthetic/natural polymers, and metals [12].

**Fig. 1:**
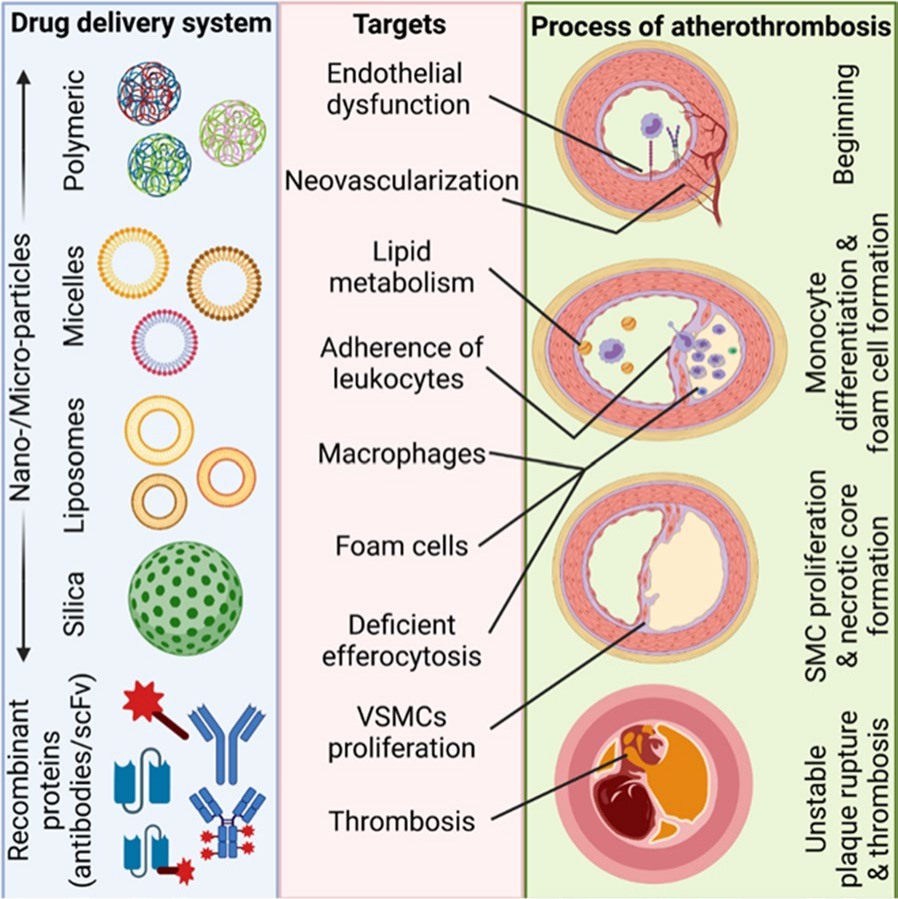
Site-targeting drugs and NPs for different stages of atherothrombosis. NPs can be made from different biomaterials and various formats of antibodies can be used to target and disrupt the atherothrombosis stages of endothelial dysfunction, neovascularization, lipid metabolism, recruitment of leukocytes, phagocytosis of macrophages, and formation of foam cells, along with inefficient efferocytosis, vascular SMC proliferation, and thrombosis

Advances in nanotechnology have led to the ability to design and generate NPs with optimal sizes (typically ranging between 1 and 100 nm), shapes, and physiochemical properties [13]. The two main advantages of using NPs are: (1) protection of the drug cargo from degradation; and (2) prevention of off-target effects and protection of the in vivo environment from potentially harmful drugs by transporting and releasing drugs at the site of disease [14]. Both biomarker-specific targeting ligands and NPs have been used for imaging and treatment of atherothrombosis (Fig. 1) [3, 15]. This review introduces the pathological mechanism of atherothrombosis and discusses recent advances in site-specific therapy using novel targeted drug-delivery and NP-carrier systems, as well as a comparison between these two strategies.

## Pathogenesis of atherothrombosis

**Fig. 2:**
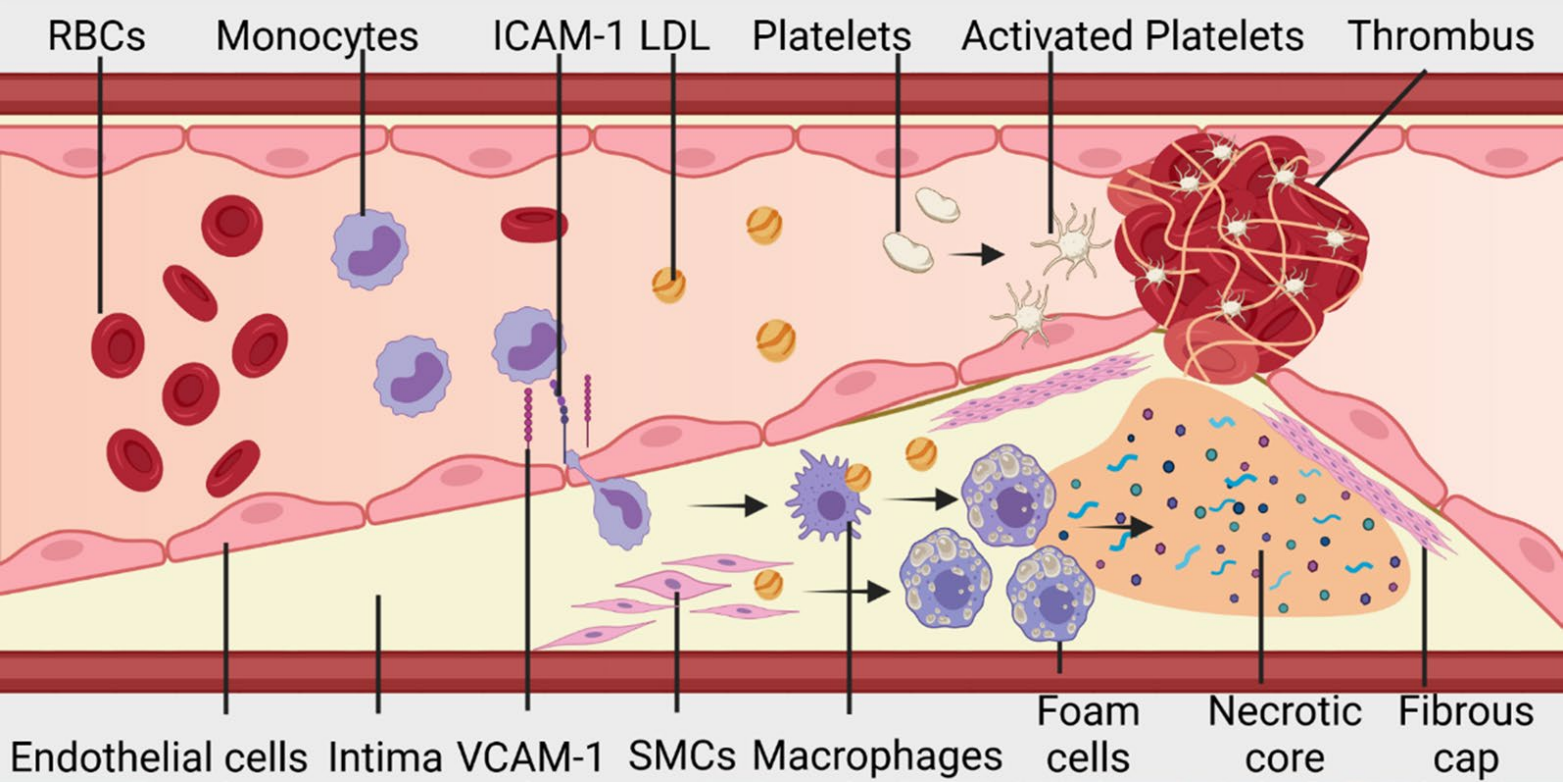
The pathology of atherothrombosis. Endothelial dysfunction causes activated endothelial cells to express inflammatory molecules, including VCAM-1 and ICAM-1. Monocytes interact with these inflammatory cell surface receptors and accumulate in the intima of blood vessels. Monocytes differentiate into lipid-loaded macrophages: LDL is taken up by macrophages and vascular SMCs leading to the formation of foam cells and the development of plaques. Accumulating and apoptotic foam cells form a necrotic core. The growing necrotic core eventually disrupts the fibrous cap and the leaking content of the plaques induces thrombosis through a series of reactions such as activation of platelets and the initiation of coagulation

Atherosclerosis is a chronic inflammatory disease characterized by lipid retention and vessel stiffening. Atherosclerosis is a complex pathological process characterized by structural abnormalities in the intima and media of arterial arteries, primarily caused by cholesterol accumulation, endothelial dysfunction, inflammatory cell infiltration, and vascular SMC migration (Fig. 2). High extracellular and intracellular lipid accumulation in advanced atherosclerotic plaques is implicated as the cause of the rupture, resulting in thrombosis and related clinical complications [16].

### The initiation of atherosclerosis

Atherosclerosis usually begins in areas of disturbed flow, typically near vessel branches and vascular bends, leading to low shear-stress recirculation, oscillation, or lateral flow [17]. Disturbed flow and its prolonged cyclic stretch on the endothelial cells have been associated with the secretion of pro-inflammatory chemokines, cytokines and high levels of reactive oxygen species (ROS) [18, 19]. These cytokines play a role in modulating endothelial cell permeability, thereby increasing the gaps between adjacent cells and compromising endothelial integrity [20]. The activation of endothelial cells leads to upregulation of a range of cell surface proteins, which in turn leads to the recruitment of leukocytes and monocytes which bind to the endothelial monolayer. Transmigration of monocytes, their infiltration of the intima and the dynamic accumulation of monocytes contribute massively towards lesion growth [20–23].

### Low-density lipoprotein accumulation and fatty streaks

Low-density lipoprotein (LDL) cholesterol is one of the most important inducers of CVDs. Cholesterol is mainly derived via intestinal absorption [24]. The liver plays a central role in its metabolism, excretion and storage [25]. Increased local and systemic levels of LDL particles invade and accumulate in the endothelium, where they are subjected to oxidation to become oxidized low-density lipoprotein (OxLDL). This OxLDL results in early fatty-streak formation, as well as further inducing endothelial dysfunction and chemokine release [20]. Reports have shown that lowering LDL from a baseline of 92 mg/dl to 43 mg/dl resulted in a decrease in cardiovascular death by 17% and further lowering to 22 mg/dl resulted in a 20% risk reduction [26, 27].

### Leukocytes and monocyte differentiation

The activation of endothelial cells leads to leukocyte recruitment by mediating monocytes rolling and promoting their adherence [28]. Depending on the cytokine signals received by monocytes, they can differentiate into dendritic cells or different phenotypes of macrophages, where classically activated (M1) macrophages has been shown to increase oxidative stress and produce pro-inflammatory cytokines [20, 29–31].

Macrophages derived from these circulating monocytes ingest the OxLDL, resulting in the formation of lipid-laden foam cells [22, 32]. The dominant pathway for foam-cell formation is mediated by scavenger receptors [33] and other independent pathways also contribute to their retention [33–36]. The accumulation of lipid-laden foam cells and immune cells contributes to the expansion of the intima and disrupts the endothelial layer, thereby promoting atherosclerotic plaque formation.

**Fig. 3:**
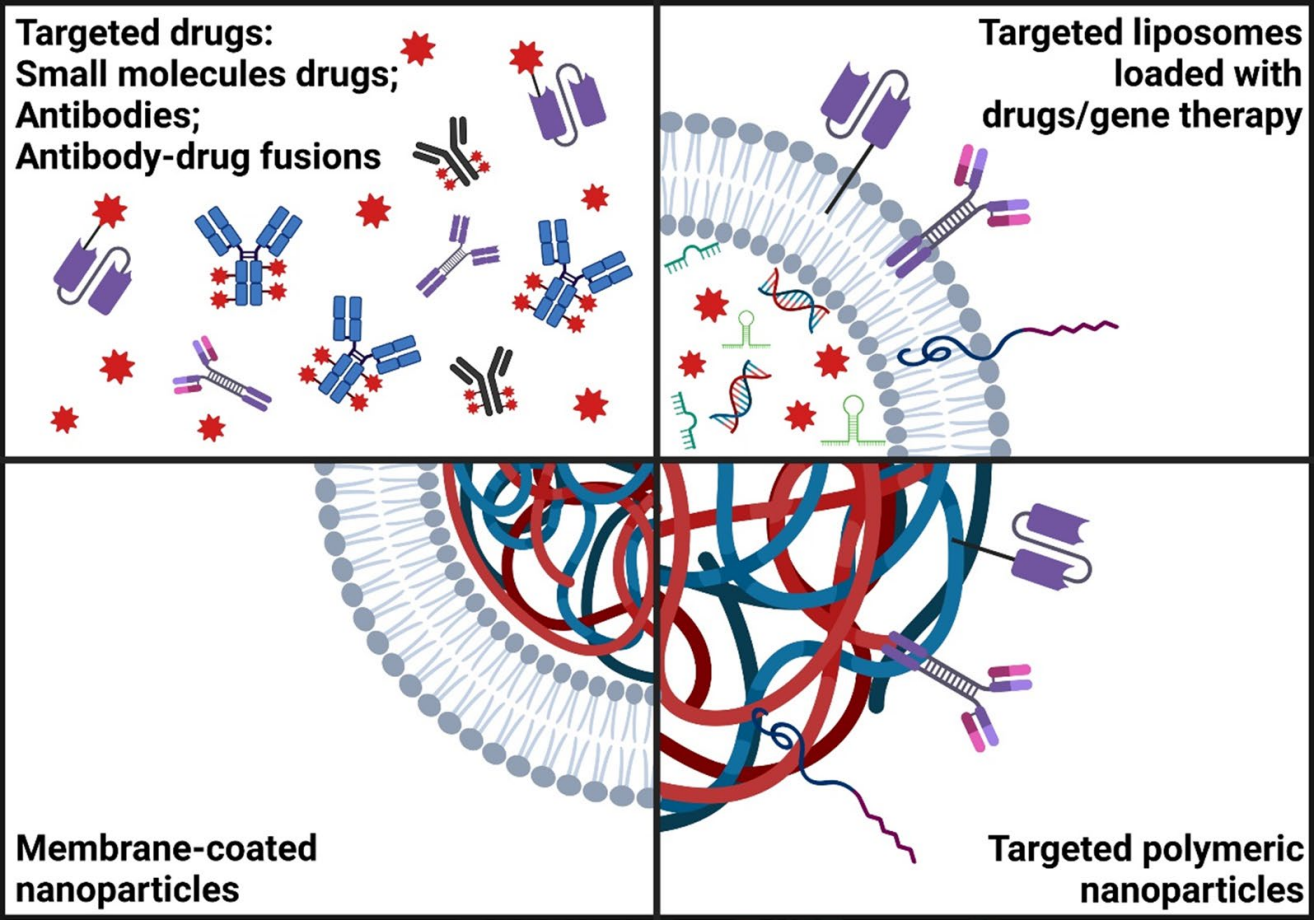
Drug and nanoparticle targeting strategies. Small molecule drugs, scFv, IgG and recombinant antibody–drug fusion complexes; liposomes conjugated with binding molecules; polymeric nanoparticles conjugated with binding molecules; and membrane-coated nanoparticles

### Proliferation of smooth muscle cells

Vascular SMCs play multiple roles in the progression of atherosclerosis [21]. During atherosclerosis, vascular SMCs undergo phenotype switching from contractile to synthetic phenotypes, resulting in these vascular SMCs to acquire the properties of macrophages and later transforming into foam cells [21, 37]. Furthermore, vascular SMCs also undergo cell proliferation, migration, and secretion of multiple extracellular matrix proteins and cytokines, all of which contribute to the formation of atherosclerotic plaques [37, 38]. Further down the track of atherosclerosis, vascular SMCs can also differentiate into osteoblast-like cells, promoting vascular calcification and aggravating plaque vulnerability [39]. It is also worth noting that vascular SMCs can play a protective role in atherosclerosis by constructing a fibrous cap which prevents rupture [40].

### Vessel remodeling and neovascularization

As foam cells accumulate and the fibrous cap thickens in the lumen, the volume of the intima enlarges, resulting in vascular remodeling and restriction of blood flow. Such lesions are usually stable but contribute to clinical discomfort. Neovascularization of atherosclerotic vessels is mainly triggered by the hypoxic regions in the thickened plaques and has been associated with lesion vulnerability [41]. These newly formed microvessels commonly arise from the adventitial vasa vasorum, in areas with increased macrophage infiltration, enhanced lipid cores, and thick cap atheroma [42].

### Unstable plaque, rupture, and thrombosis

Although luminal narrowing caused by an atherosclerotic plaque contributes to some clinical symptoms, most acute and severe clinical manifestations are triggered by atherothrombosis [43]. Three different morphological entities have been recognized as the etiology of coronary thrombosis: plaque erosion, calcified nodules, and plaque rupture. Plaque erosion usually occurs in lesions with high levels of proteoglycans and SMCs because their disruption of endothelial integrity exposes the underlying collagen, which becomes a nidus for thrombosis [44]. Calcified nodules occur primarily in older patients when disruption of calcified necrotic cores triggers thrombosis [45]. Rupture of the fibrous cap on an unstable or vulnerable atherosclerotic plaque leads to exposure of the necrotic core content such as tissue factors (TFs) to the flowing blood and they activate a coagulation cascade [46].

In summary, atherothrombosis is a complex disease which contributes to one in three deaths. Current pharmacological treatments have been associated with drug resistance and side effects [8, 47]. Therefore, site-specific targeted drugs and the use of NPs as a controlled carrier system have been used to overcome the need for high systematic doses and the problem of bleeding complications.

## Site-specific drug delivery and nanoparticles

The site-specific delivery of drugs has been widely researched in all medical fields. The use of NPs as drug-delivery strategies has been widely accepted in cancer therapy, with the first nanotherapeutic, Doxil, approved for clinical use in 1995 [48, 49]. Furthermore, NPs were clinically approved for drug therapy for acute myeloid leukemia in 2017 and for gene therapy for transthyretin-mediated amyloidosis in 2018 [48, 50]. The most recent clinical success in NP use is the two COVID-19 messengerRNA (mRNA)-based vaccines to combat severe acute respiratory syndrome coronavirus 2 (SARS-CoV-2) [51–55]. The successful use of nanomedicine in these medical areas has inspired many cardiovascular researchers to harness NPs’ inherent properties such as their passive diffusion and endocytosis for in vitro and in vivo experiments. Passive accumulation can lead to unwanted off-target delivery; therefore, a range of conjugation techniques have been established to enable active targeting to specific biomarkers of CVDs or trigger drug release by responding to biological or technical stimuli. Since CVDs are complex diseases, the most difficult aspect of developing site-specific therapies is the ability to identify an individual and unique antigen that is only expressed in lesions or diseased areas. In the following sections, we will discuss some of these unique targets that are important or upregulated during the progression of atherosclerosis and atherothrombosis, as well as the research on the use of targeted drugs and NPs for drug delivery (Fig. 3).

## Strategies for targeting atherothrombosis

### Endothelial cells

**Fig. 4:**
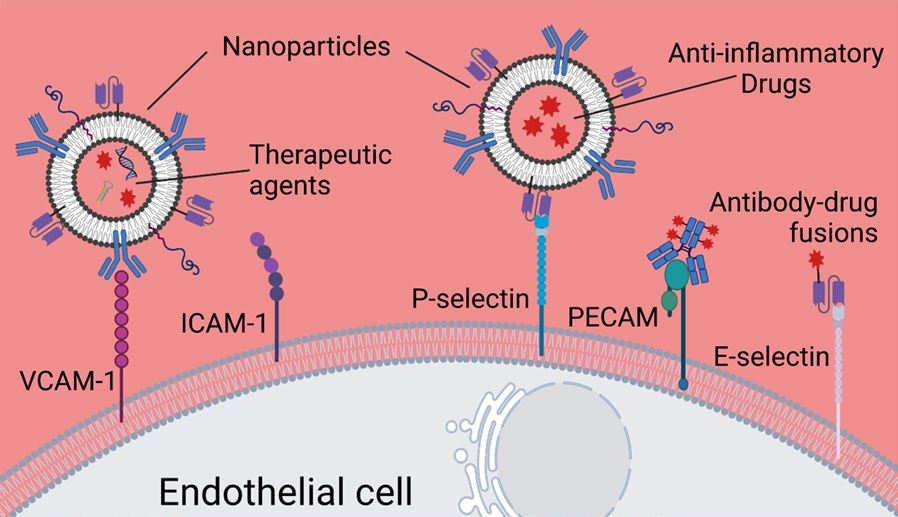
Targeted delivery to inflamed endothelial cells. Adhesion molecules expressed by endothelial cells during inflammation include VCAM-1, ICAM-1, PECAM, E-selectin, and P-selectin. Anti-inflammatory therapy includes the direct targeting and blockage of these adhesion molecules using antibodies, as well as nanoparticles for the delivery of pharmaceutic and genetic agents

Vascular endothelial cells play several essential roles, including maintaining vascular homeostasis, regulation of vascular tension, proliferation of SMCs, and prevention of inflammation and thrombosis [56]. However, the activation of endothelial cells leads to the secretion of pro-inflammatory chemokines and cytokines, such as interleukin (IL)-8 and monocyte chemotactic protein-1 (MCP-1), and high levels of ROS [18, 19, 57, 58]. The activation also results in the upregulation of a range of cell surface proteins, including vascular cell adhesion molecule-1 (VCAM-1), intercellular cell adhesion molecule-1 (ICAM-1), P-selectin, and platelet endothelial cell adhesion molecule-1 (PECAM-1) (Fig. 4). Since endothelial dysfunction is the initial step in atherosclerosis, endothelial cells and their cell adhesion molecules have been intensively explored as crucial drug-delivery targets to prevent or slow the progress of atherosclerosis (Table 1) [59, 60].

**Table 1:** Antibodies and NPs targeting endothelial cells

Targeting approach	Delivered drugs	Models	Effect	Refs.
PLGA NP + GPIb fragment	Dexamethasone	In vitro HAECs and ex vivo carotid artery of mice	GPIb-conjugated NPs had five-fold greater absorption by HAECs	[81]
DOPE-liposome	CD39 mRNA	In vitro A549 cell	Induced a significant change in CD39 expression	[84]
Anti-VCAM-1 mAbs	Anti-VCAM-1 mAbs	ApoE^−/−^ on HFD (mice)	Inhibited vascular inflammation	[61]
Cationic liposome + VHPK	miRNA-712	Carotid ligation (mice)	Prevented atheroma development	[62]
MB + anti-VCAM-1 scFv	miRNA-126	Ang II induced AAA (mice)	Changed vessel diameter in AAA murine model	[63]
Liposomes + E-selectin–binding peptide	Atorvastatin calcium & *Curcuma longa*	ApoE^−/−^ on HFD (mice)	Inhibited E-selectin and ICAM-1 expression, lowered plasma lipid, prevented foam-cell formation and secretion of inflammatory factors	[72]
Copolymer + E-selectin–binding peptide	Dexamethasone	ApoE^−/−^ on HFD (mice)	Regressed and stabilized atherosclerotic plaques	[73]
Microparticles + E-selectin–binding thioaptamer	miR145a & miR181b	ApoE^−/−^ on HFD (mice)	Decreased plaque size and macrophage infiltration in the aortic root	[74]
Lipid nanoemulsions conjugated to E-selectin–binding peptide	Dexamethasone	C57BL/6 (mice)	Significantly decreased expression of pro-inflammatory markers and endothelium activation, as well as monocyte infiltration	[79]
P-selectin-targeted cationic PEGylated liposome	RAGE-short hairpin (sh) RNA	ApoE^−/−^ on HFD (mice)	Inhibited leukocytes recruitment and subsequent atherosclerosis	[80]
Peptide analogue IELLQAR	Selectin inhibition	ApoE^−/−^ on HFD (mice)	Inhibited selectin binding to monocytes and subsequent atherosclerosis	[75]
Oligopeptide-modified pBAE NP	siRNA	C57BL/6 (mice)	Tissue-driven targeting with high affinity to the artery endothelium, lung, and kidney	[87]

#### Vascular cell adhesion molecule-1

Of the different biomarkers upregulated on inflamed or dysfunctional endothelial cells, VCAM-1 results in leukocytes rolling/binding and is the most promising target [18, 61]. Therefore, anti-VCAM-1 monoclonal antibodies (mAbs) have been investigated as a therapeutic agent for atherosclerosis. Park et al. developed mAbs against VCAM-1 (H6 and 7H) via phage display and found success in inhibiting atherosclerosis in vivo using a traditional apolipoprotein-E–deficient (ApoE^−/−^) atherosclerosis murine model. The study also showed that anti-VCAM-1 mAbs reduced ROS generation and RhoA activation in endothelial cells and prevented lymphocyte transmigration by binding to the extracellular domains 1–2 of VCAM-1 [61]. Therefore, blocking of VCAM-1 can directly reduce leukocyte recruitment and inflammation.

In addition to direct anti-VCAM-1 blockage, groups have employed NPs and microparticles for targeted gene therapy [62, 63]. By altering gene expressions during CVDs, these studies have prevented the progression of atherosclerosis and rupture of aneurysms [62, 63]. Since regions with disturbed blood flow initiates the occurrence of atherosclerosis, studies have shown the upregulation of most flow-sensitive microRNA (miR)-712 [64, 65]. Increased levels of miR-712 directly downregulate tissue inhibitors of metalloproteinase-3, which in turn contribute to the proliferation and migration of SMCs [65–67]. Kheirolomoom et al. designed a VCAM-1–targeting cationic liposome to deliver anti-miR-712 to endothelial cells using a peptide sequence of VHPKQHR (VHPK), which is a VCAM-1–internalizing sequence [62]. This study demonstrated the selective uptake of the particles into the endothelium and the inhibition of atherosclerosis in vivo using a traditional ApoE^−/−^ murine atherosclerosis model [62].It is worth noting that anti-miR-712 was only internalized by the surface endothelial cells and was not further transported into the vessel wall, which might indicate that the NPs are too large to travel through the endothelial layer.

**Fig. 5:**
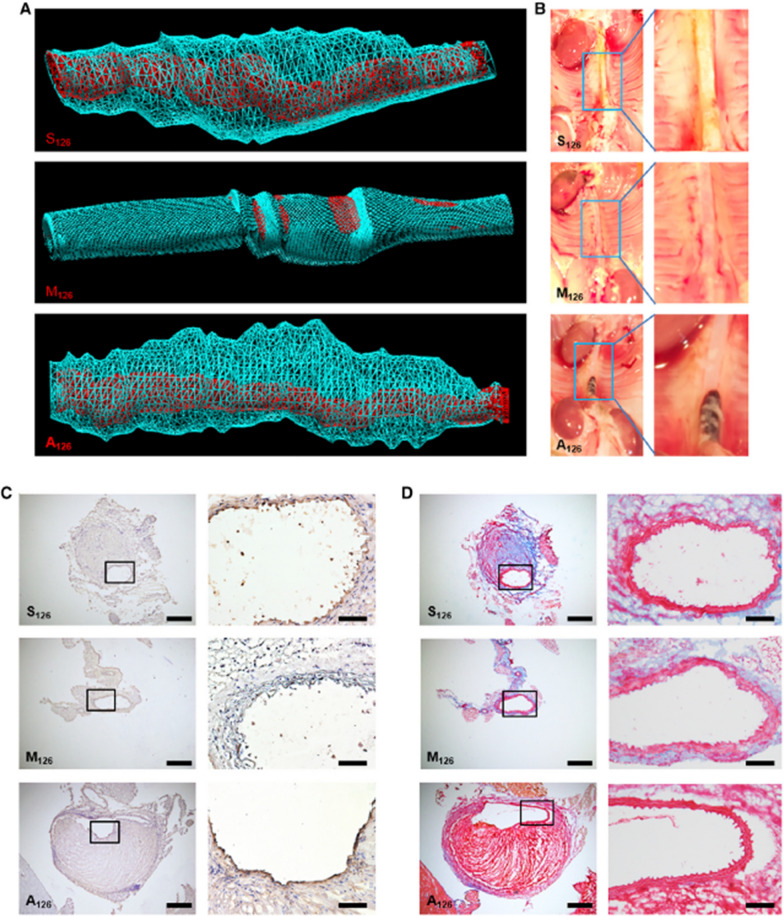
Representative images of 3D ultrasound reconstructions of abdominal aorta, photographs of vessel isolations, immunohistochemistry, and Martius Scarlet Blue demonstrating profound effect of VCAM-1–targeted miR-carrying microbubbles.** A** 3D ultrasound reconstruction of abdominal aorta shows vessel lumen (in red), as well as massive areas of plaque build-up and aneurysm (in blue), from animals treated with Targ^MB^-A_126_ or Targ^MB^-S_126_ but not in animals treated with Targ^MB^-M_126._ **B** Vessel isolation shows clean abdominal aorta in mice treated with Targ^MB^-M_126_ but plaque build-up and aneurysms in mice given Targ^MB^-S_126_ or Targ^MB^-A_126_. **C** Immunohistochemistry confirmed a decrease in VCAM-1 expression for Targ^MB^-M_126_ treated animals as compared to those treated with Targ^MB^-A_126_ or Targ^MB^-S_126_. **D** Martius Scarlet Blue showed plaque build-up and aneurysms in abdominal arteries of Targ^MB^-A_126_ or Targ^MB^-S_126_ treated animals, whereas very little plaque build-up was observed in Targ^MB^-M_126_ treated mice [63]

Abdominal aortic aneurysm (AAA), which has a frightful mortality rate of up to 90%, if undetected and left untreated [2, 68]. AAA’s pathological characteristics exhibit close similarities to atherosclerosis, where strong inflammatory and thrombotic processes are driving forces of both diseases [69]. Wang et al. designed theranostic particles for concomitant diagnosis and therapy [63]. The group used a single-chain variable fragment antibody (scFv) that targets VCAM-1 to direct their microbubbles (MBs) (the ultrasound contrast agent) toward the inflamed endothelial cells on the vessels in an angiotensin II-induced AAA murine model [63]. The authors then coated the VCAM-1–targeted MBs with miR-126-5p, which has been shown to inhibit VCAM-1 expression and atherosclerosis [70]. To prevent off-target effects, the group also applied ultrasound pressure to the abdominal artery to burst the MBs and deliver the miR-126 into the endothelial layer [63]. After 4 weeks of dual-targeted treatment, the vessel diameter of the AAA murine model remained unaffected by angiotensin II induction, whereas there was an increase in vessel diameter in the control groups [63]. There were also massive areas of plaque buildup and ruptured aneurysms in control groups which were not observed with the dual-targeted treatment (Fig. 5) [63]. Since MBs have already been approved by the US Food and Drug Administration (FDA), the clinical translation of this technique may be substantially faster than with unapproved, newly generated particles. The development of AAA, including the intraluminal thrombosis is very similar to the pathogeneses of atherosclerosis and atherothrombosis. These diseases share multiple common mechanisms, such as the destabilization of the vessel wall and endothelial dysfunction [69, 71]. Wang et al.’s dual targeted approach employing theranostic particles for AAA may also initiate the development of theranostic strategies for atherothrombosis.

#### Endothelial-leukocyte cell adhesion molecule (E-selectin)

E-selectin is expressed on inflamed endothelial cells upon their activation via cytokines. Using liposomes conjugated with an E-selectin–binding peptide, Li et al. performed targeted co-delivery of hypolipidemic and anti-oxidation drugs in vivo and observed atherosclerosis inhibition in a traditional ApoE^−/−^ murine atherosclerosis model [72]. The authors used atorvastatin calcium to reduce plasma lipid and Curcuma longa, which has anti-oxidation and anti-inflammatory properties [72]. Their therapeutic liposomes inhibited the expression of E-selectin and ICAM-1 in vitro and prevented foam-cell formation in vivo. The levels of plasma lipid and expression of inflammatory factors (IL-6 and MCP-1) were also lower in treated mice [72]. Although the study authors also mentioned that their targeted therapy might reduce the current side effects of atorvastatin calcium, such as central nervous system complaints, CoQ10-lowering effects, and diabetic mellitus, these data were not presented.

Another study by Tsoref et al. employed an E-selectin–binding peptide to deliver an anti-inflammatory drug (dexamethasone) by packaging it into polymer-based NPs [73]. These targeted drug-loaded NPs resulted in the regression and stabilization of atherosclerotic plaques, as well as the prevention of left ventricular remodeling in ApoE^−/−^ mice [73]. Using the E-selectin thioaptamer conjugated on nanoporous silicon microparticles to deliver atheroprotective miR-145a and miR181b, Ma et al. successfully decreased plaque size and macrophage infiltration in the aortic root of ApoE^−/−^ mice [74]. The study also showed increased numbers of SMCs and collagen content in plaque areas, resulting in a reduction in atherosclerosis in vivo [74]. Overall, these studies indicated that E-selectin and VCAM-1 are ideal biomarkers for targeted drug and gene delivery towards inflamed endothelial cells.

#### P-selectin

P-selectin is expressed on damaged endothelium during inflammation. A recent study by Ye et al. investigated the use of a peptide analogue that inhibits P-selectin binding and demonstrated a reduction in the formation of atherosclerotic plaques and infiltration of monocytes/macrophages in the arterial wall of ApoE^−/−^ mice [75]. Other groups have used the sialyl LewisX (sLeX) carbohydrate to target P-selectin and demonstrated reduced monocyte adhesion and endothelium injury [76]. Using a combination of sLeX and a P-selectin–inhibiting antibody, Ikeda et al. showed that this therapy prevented the adherence of platelets to leukocytes at the stenotic sites on the damaged endothelium in a canine model [77]. Using liposomes conjugated with the P-selectin glycoprotein ligand 1, these P-selectin–targeting particles resulted in greater than sevenfold binding affinity toward activated epithelial cells in vitro [78]. Nanoemulsions loaded with dexamethasone and conjugated to P-selectin–binding peptides demonstrated accumulation in the lungs of mice in a lipopolysaccharide-induced systemic inflammation model [79]. These targeted particles significantly decreased the expression of pro-inflammatory markers and endothelium activation, as well as monocyte infiltration, thereby reducing the inflammation in the lungs [79]. A recent study by Mocanu et al. demonstrated that P-selectin-targeted liposomes for transfection of short hairpin RNA, encoded for RAGE, resulted in the downregulation of RAGE expression and the prevention of atherosclerotic plaques in ApoE^−/−^mice [80]. Expression of P-selectins on endothelial cells also triggers binding by the platelet glycoprotein Ib-alpha (GPIbα). Therefore, Kona et al. developed poly(lactic-co-glycolic acid) (PLGA) NPs conjugated with glycocalicin, which is the external soluble cleaved fragment of platelet GPIbα, to deliver dexamethasone [81]. These targeted biodegradable NPs had an increased uptake by human aortic endothelial cells in vitro and in the carotid artery of mice ex vivo [81]. However, since P-selectins are upregulated on both endothelial cells and activated platelets, there is a need for further in vivo studies to determine their biodistribution and in vivo therapeutic effect, as well as to investigate any possible complications.

#### Novel genetic therapy approaches for endothelial dysfunction

Many groups have investigated gene therapy targeting endothelial dysfunction in recent years due to its involvement with inflammation. Novel NPs have been created to transport and protect nucleic acids during their delivery. The delivery of mRNA is a challenge because it can be easily degraded by nucleases and is intrinsically unstable [82]. Therefore, Michel et al. developed a nanoliposomal system using 3β-[N-(N’, N’-dimethylaminoethane) carbamoyl] (DC-cholesterol)/dioleoyl-phosphatidyl-ethanolamine (DOPE) nanoliposomes to protect the mRNA and facilitate their delivery into the cells, and this provided a long-lasting therapeutic effect in vitro [83]. These nanoliposomes have also been reported to be highly hemocompatible and biocompatible in vitro [83]. CD39, a nucleoside triphosphate diphosphohydrolase (NTPDase), is expressed on endothelial cells and has anti-inflammatory and anti-thrombotic effects. Using CD39 coding mRNA, Abraham et al. demonstrated that their nanoliposomes exhibited a high degree of mRNA encapsulation and high efficiency of transfection in vitro [84]. CD39 hydrolyzes both the pro-inflammatory adenosine triphosphate (ATP) and pro-thrombotic adenosine diphosphate (ADP) to the anti-inflammatory and anti-thrombotic adenosine monophosphate (AMP). Furthermore, mice lacking CD39 always exhibit a pro-thrombotic and pro-inflammatory response from endothelial cells, with increased production of ICAM-1 and VCAM-1, as well as the release of TNF-α and vWF, and decreased anti-inflammatory mediators [85]. However, these transgenic mice also exhibited impaired platelet function and an increase in bleeding time [86]. Therefore, this successful in vitro study of CD39 mRNA requires in vivo investigation to determine its suitability as a potential therapy.

To overcome the interaction of NPs with serum lipoproteins and their passive accumulation in the liver, Dosta et al. developed a series of oligopeptide-modified poly-beta-amino ester (pBAE) NPs to deliver small interfering RNA (siRNA) against ICAM-2 in vivo [87]. The group chose pBAE because it is a biodegradable and biocompatible polymer that can penetrate cell membranes easily [88]. While the final choice of the group named C6-KH was selected for in vivo studies, all 7 pBAEs bound to endothelial cells, vascular SMCs, and THP-1 monocytes to some degree [87]. Ex vivo studies using the abdominal aortas of mice showed binding of these NPs to vessel walls, while in vivo biodistribution studies showed a significant reduction in ICAM-2 levels in the lungs and the kidneys, but a slight increase in the spleen and no difference in the thymus, liver, or heart [87]. While the study did not explain why these NPs would accumulate and downregulate ICAM-2 expression in the lungs and kidneys of the healthy animals, it may be due to non-specific uptake of NPs by the reticuloendothelial system. Thus, in vivo studies need to be completed to determine their specificity to avoid off-target effects, and successful application as a therapeutic strategy. Overall, NPs protect the nucleic acids from degradation in vivo and the functionalization of these NPs to target specific biomarkers will result in site-specific therapy and minimize the off-target effects.

### Leukocytes

Adhesion molecules, particularly leukocyte ß_2_-integrins, mediate the recruitment of leukocytes to the inflamed endothelial layer [89]. The macrophage-1 (Mac-1) antigen (α_M_ß_2_, CD11b/CD18, complement receptor 3) is a ß_2_-integrin family member that is abundantly expressed on monocytes and neutrophils [90]. Its ectodomains can be stimulated to an active conformation by cellular activation, allowing it to interact with various ligands such as fibrinogen, C3bi, ICAM-1, and heparin [91]. In a recent study, a designed ankyrin repeat protein (DARPin) that specifically targets the ß_2_-integrin Mac-1 successfully prevented Mac-1 activation [92]. Siegel et al. also showed that the Mac-1–specific DARPin impeded vascular inflammation via significantly reduced monocyte binding with ICAM-1 and demonstrated advantageous cross-reactivity between human and mice monocytes and neutrophils [92]. This new compound also effectively prevented leukocytes from migrating to the peritoneal lavage from blood, inhibited monocyte–cardiomyocyte interactions, and improved myocarditis and cardiac ischemic/reperfusion (I/R) injury in vivo [92]. This DARPin may be a novel tool that inhibits inflammation by preventing interaction between leukocyte ß_2_-integrins and adhesion molecules, as well as other downstream therapies. The use of DAPRins has several benefits, 1) their small size allows better tissue penetration and high potency at low concentrations, 2) microbial expression allows for large scale production, and 3) they are thermodynamically stable.

While the inhibition of leukocytes adhesion has been proven successful in preventing atherosclerosis and endocytosis of phagocytes, it can also be harnessed to transport NPs to inflamed lesions and plaques through cellular recruitment and infiltration [93]. To reduce ROS levels, Wang et al. created NPs using cyclic polysaccharide β-cyclodextrin, which removes cholesterol, and phenylboronic acid pinacol ester, which is a catalase mimetic chemical to remove hydrogen peroxide effectively [107]. Using flow cytometry, the authors demonstrated that triple-drug-loaded NPs were taken up by lymphocytes, neutrophils, and Ly-6C^low^ and Ly-6C^high^ monocytes, as well as macrophages and SMCs in vivo [94]. Furthermore, in vivo studies using traditional ApoE^−/−^ mice after treatment with triple-drug-loaded NPs showed stabilized plaques, along with fewer macrophages and lower levels of ROS, TNF-α, IL-1β, and MMP-9 as compared with mice treated with control drugs [94]. The therapeutic benefits mainly resulted from reduced systemic and local oxidative stress and inflammation, as well as decreased inflammatory cell infiltration in atherosclerotic plaques [94]. While the use of these leukocyte markers have been successful in reducing inflammation, these therapies have also been shown to be beneficial in other diseases, such as sepsis [92, 95]. Therefore, more in vivo studies need to be conducted to ensure that the targeting and direct inhibition of leukocytes do not affect other immunological responses in the body.

### Macrophages

Phagocytosis of OxLDL makes macrophages one of the major contributors to plaque development; this phagocytic property also allows them to engulf non-targeted NPs more efficiently than monocytes [94, 96]. Therefore, many researchers have exploited the passive accumulation of drug-loaded NPs in macrophages as an approach to disrupting atherosclerosis (Table 2).

**Table 2:** NPs targeting macrophages

Targeting approach	Delivered drugs	Models	Effect	Refs.
Amphiphilic macromolecule (AM)		In vitro SMCs	Prevented LDL internalization in SMCs and reduced MSR1 and CD36 expression	[108]
AM (1 cM)	Ferulic acid	In vitro human monocyte– derived macrophages	Reduced uptake of oxLDL and ROS level	[103]
Scorpion-like AM	Lithocholic acid	In vitro human monocyte– derived macrophages	Lowered oxLDL uptake by macrophages	[107]
PLA-PEG NP	Rosiglitazone (RSG)	In vitro RAW264.7 macrophages	Significantly inhibited IL-10 expression	[121]
rHDL NP	Statin	ApoE^−/−^ on HFD (mice)	Accumulated in macrophages and decreased inflammation and lower inflammatory factors	[96, 100]
AM	GW3965	HFD (rats)	Lowered intimal levels of accumulated cholesterol, inhibited macrophage retention	[105]
Sugar-based AM (M_12_PEG) against MSR1 & CD36 SR		ApoE^−/−^ mice on Harlan Teklad diet	Reduced artery occlusion, inhibited uptake of oxLDL, downregulated expression of SRs	[106]
Mannose-functionalized NPs	LXR ligand	LDLR^−/−^ on HFD (mice)	Reduction of lesion area in atherosclerotic plaques	[138]
Hyaluronan NPs		ApoE^−/−^ on HFD (mice)	Lowered number of immune cells in plaques	[111]
	LOX-1 siRNA	ApoE^−/−^ on HFD (mice)	Reduced plaque area and lipid content	[113]
	Simvastatin	ApoE^−/−^ on HFD (mice)	Significantly reduced plaque size	[114]
Cationic peptide containing pH-sensing residues (p5RHH)	JNK2 siRNA	ApoE^−/−^ on HFD (mice)	Decreased thrombotic risk, restored endothelial barrier integrity, reduced plaque necrosis, and depleted plaque-macrophage content	[115]

#### Reconstituted high-density-lipoprotein–based drug-delivery systems

Reconstituted high-density lipoproteins (rHDLs) are atheroprotective because they interact with ATP-binding cassette transporters (ABCA1, ABCG1) and the scavenger receptor B1 to remove cholesterol from lipid-laden macrophages [97–99]. Using rHDL as a basis for PLGA NPs, Sanchez-Gaytan et al. demonstrated their accumulation in the plaques of ApoE^−/−^ atherosclerotic mice, as well as their co-localization with macrophages [100]. By combining rHDL NPs with a lipid-lowering drug, statin [101], Duivenvoorden et al. demonstrated their potential in reducing plaque inflammation and inhibiting plaque progression in vivo [96]. The authors observed that intravenously delivered statin-loaded rHDL NPs increased the bioavailability and infiltration of statin in atherosclerotic plaques [96]. The mRNA expression levels of monocyte-recruitment genes (MCP-1, CCL-3, ICAM-1, VCAM-1, CCL-15, and CXCL-12), as well as pro-inflammatory genes (TNF-a, IL-1b, IL-1a, and SPP-1), were significantly lower in the treated mice as compared to the control mice treated with statin orally [96]. While more in vivo studies are needed to further evaluate the efficacy and biodistribution of these NPs, it is worth noting that HDL is a natural atheroprotective lipoprotein and is currently in Phase 3 clinical trials (ClinicalTrials.gov – NCT03473223) [102]. Therefore, the use of HDL as a building block for NPs and its combination with lipid-lowering drugs may provide an attractive therapeutic approach for atherosclerosis.

#### Amphiphilic macromolecule–based drug-delivery systems

The Moghe group has developed a series of amphiphilic macromolecules (AMs) to deliver a range of therapeutics to macrophages using amphiphilic polymers containing a carboxylic acid group that binds scavenger receptors of macrophages, thereby inhibiting the uptake of oxLDL [103–107]. Using sugar-based AMs that bind scavenger receptors of macrophages, Chan et al. discovered that they could prevent up to 90% of LDL internalization in SMCs and inhibit up to 48% of macrophage scavenger receptor 1 (MSR1) expression and 33% of CD36 expression in an in vitro study [108].

Polyphenol compounds, such as vitamin E and ferulic acid, have been utilized in therapies for atherosclerosis because of their hypolipidemic, anti-inflammatory, and anti-oxidative effects [103, 109]. However, a high concentration of antioxidants plays a reverse role, causing oxidative stress, and may result in exacerbation of foam-cell formation in lesions [110]. Therefore, a system that directs the delivery of antioxidants to atherosclerosis sites and provides slow release is preferred. Chmielowski et al. incorporated ferulic acid into these AMs to counteract oxLDL uptake and regulate ROS generation, limiting macrophage foam-cell formation [103]. Moretti et al. incorporated lithocholic acid, an agonist against the G-protein coupled receptor 19 that is known to reduce inflammation, and demonstrated a slight downregulation of scavenger receptors and pro-inflammatory cytokine (IL-1β, IL-8, and IL-10) expression [107]. However, both studies were in vitro; therefore, further studies need to be conducted to determine their efficacy in vivo. Using sugar-based AMs, Lewis et al. demonstrated that they accumulated at atherosclerotic lesions, where they reduced plaque formation and lesion severity in vivo [106]. The group has chosen to administer four therapeutic doses over the 8 weeks experimental period; however, the rationale for weekly injections only in the first 4 weeks was not clear. There is a need to investigate and better define the time point for administration according to the biodistribution and therapeutic effects of these AMs.

#### Hyaluronic acid–generated particles for drug delivery

Many research groups have investigated the atheroprotective effect of particles generated by hyaluronan, or hyaluronic acid (HA). Beldman et al. demonstrated via PET imaging that radiolabeled HA–NPs accumulated at the sites of atherosclerosis in ApoE^−/−^ mice and resulted in reduced macrophages in the plaque lesions [111]. Interestingly, the authors showed that the uptake of HA–NPs by macrophages was sixfold more effective in early lesions than in advanced plaques [111]. This finding may be due to higher endothelial permeability remodeling in early lesions as compared to advanced plaques, where there are increased collagen and SMC proliferation [112]. Using HA–coated nanocomplexes decorated with lectin-like oxidized low-density lipoprotein receptor (LOX-1) siRNA cell-penetrating peptides, Zhao et al. demonstrated reductions in plaque area by 28% and neutral lipid content by 37%, as well as less infiltration of macrophages and chemokines in ApoE^−/−^ mice [113]. Mu et al. loaded simvastatin into biodegradable HA-coated polymeric micelles and found that treatment with these particles resulted in significantly reduced plaque size in ApoE^−/−^ mice on HFD [114]. In these studies, HA may have also played an anti-oxidative role further improving the observed therapeutic effects.

#### Perfluorocarbon NPs for gene delivery

Pan et al. created pH-sensing cationic perfluorocarbon NPs and coated them with siRNA against the Jun N-terminal kinase (JNK), a stress-activated protein kinase that mediates proapoptotic responses following stimulation by environmental stresses, cytokines, and growth factors. The authors demonstrated that these particles reduced necrosis and depleted plaque macrophage content in the plaques of ApoE^−/−^ mice after seven doses of treatment [115]. The group also showed a reduction in the time to occlusion in acute thrombosis in the treated mice and attributed this to the NPs’ ability to restore the integrity of the endothelial barrier [115]. The choice of perfluorocarbon is interesting because it was previously used as blood substitute and may thus allow a smooth transition into their clinical use. These perfluorocarbon NPs are currently used as 19-Fluorine MRI contrast [116, 117] and ultrasound contrast agents [118], therefore moving forward, these could potentially be used in theranostic approaches.

#### Peroxisome proliferator–activated receptor gamma agonist

The peroxisome proliferator–activated receptor gamma (PPARγ) is a member of the ligand-inducible transcription factor family that regulates glucose homeostasis and is highly expressed in macrophage foam cells [119]. Using two distinct PPARγ agonists, rosiglitazone and GW7845, both of which are insulin sensitizers for the treatment of diabetes, Li et al. successfully limited the development of atherosclerosis in LDLR^–/–^ mice [119]. In this study, these agonists upregulated CD36, but the treated mice also had improved insulin sensitivity and reduced inflammatory markers [119]. This is likely because PPARγ is involved in the regulation of various immunological events, including the differentiation and activation of immune cells, and the regulation of cytokine expression, all of which contribute to remodeling of the immune balance [120]. To overcome the high systemic dose required and off-target effects of such agonists, Giacalone et al. encapsulated rosiglitazone into polymeric NPs and demonstrated that they inhibited inflammatory cytokine (IL-10) expression in RAW264.7 macrophages [121]. This study revealed the potential of PLA-PEG-RSG NPs to suppress the inflammatory response of atherosclerosis. However, more research is needed to enhance these NPs’ targeting capacity and test their efficacy in vivo.

### Efferocytosis

Effective efferocytosis and removal of apoptotic cells reduce plaque cellularity and suppress the growth of plaques, whereas ineffective efferocytosis results in the accumulation of apoptotic debris and formation of a necrotic core [122–124]. An interesting approach to avoiding the inflammatory repercussions associated with the accumulation of apoptotic cells is to block cell-surface expression of anti-phagocytic signals like CD47. Kojima et al. demonstrated that therapy using an inhibitory antibody directed against CD47 which can increase the effectiveness of efferocytosis was critical for reversing the buildup of foam cells and reduced plaques in vivo [125]. Although the anti-CD47 antibody can offer a new way to increase efferocytosis, this treatment has to be used with caution because the antibody may result in side effects such as erythrophagocytosis and anemia [126].

### Lipid metabolism

Elevated LDL levels in familial hypercholesterolemia are linked to three single genetic mutations: LDL receptors, proprotein convertase subtilisin/kexin type 9 (PCSK9), and apolipoprotein (Apo) B [127].

#### Promoting lipid storage in liver

PCSK9, a member of the proteinase K subfamily of subtilases, is a ligand of LDL receptors, causing their degradation in the lysosome [127]. The absence of PCSK9 has been associated with increased expression of LDL receptors, a phenomenon that can be altered with the enhancement of PCSK9 via mRNA gene therapy [128]. In a phase 1 trial, Fizgerald et al. demonstrated that the encapsulation of siRNA against PCSK9 via a lipid NP resulted in a 70% reduction in circulating PCSK9 and 40% reduction in LDL in patients as compared with the placebo group [129]. The downregulation of PCSK9 resulted in higher expression of LDL receptors and more efficient cholesterol absorption, thereby lowering plasma cholesterol levels, and was an effective therapy. A limitation for this approach is the need for multiple injections, hence an increase in cost and health care burden.

Since the minimal promoter region of the PCSK9 gene houses a sterol-regulatory element (SRE), the transcription of PCSK9 is dependent on SRE-binding proteins (SREBPs). The silencing of these proteins interferes with the PCSK9–LDL receptor pathway, as well as cholesterol biosynthetic activity in the liver, which may help to manage plasma cholesterol levels more effectively. Since NPs passively accumulate in the liver, many research groups have harnessed this property to achieve or enhance their therapeutic strategy for lipid metabolism (Table 3) [130]. SREBPs regulate lipid homeostasis by modulating the genes which control lipid biosynthesis via altering the expression of LDL receptors and the PCSK9 gene. The inhibition of the SREBP cleavage-activating protein (SCAP) resulted in the reduction of circulating PCSK9, LDL, and triglycerides. In a rhesus monkey model, spontaneous dysmetabolic gene therapy, using siRNA against SCAP and lipid-based NPs, decreased PCSK9, LDL, and triglycerides [131, 132]. Similar results were observed via targeting simvastatin with a monoclonal antibody with high affinity to PCSK9 with simvastatin in both mice and rhesus monkeys [10]. In another gene-therapy approach, nanoliposomes with siRNA against ApoB successfully lowered plasma cholesterol levels and expression of LDL receptors in hepatic cells in vivo [133].

**Table 3:** NPs targeting lipid metabolism

Targeting approach	Delivered drugs	Models	Effect	Refs.
Lipid NPs	PCSK9 siRNA	Phase 1 trial (humans)	Triggered a mean 70% reduction in circulating PCSK9 and 40% reduction in LDLC in patients compared with placebo group	[129]
Lipid NPs	SCAP siRNA	Dysmetabolic model (Rhesus monkey)	Reduced circulating LDLC, PCSK9, and TG	[131, 132]
Anti-PCSK9 monoclonal antibody (1B20)	1B20/simvastatin	LDLR^±^ mice and Rhesus monkeys	Significant reduced plasma LDLC	[10]
Lipid NPs	ApoB siRNA	LDLR^+/–^ CETP^+/–^ mice model on HFD	Downregulated expression of ApoB and thus reduced serum lipid levels	[133]
SeNPs	Selenium	ApoE^−/−^ mice on HFD	Decreased serum TC and TG levels	[135, 137]

#### Preventing lipid oxidation

Another compound used as an anti-atherosclerotic agent is selenium, because it has been shown to prevent oxidative modification of lipids, suppress platelet aggregation, and reduce inflammation [134]. However, elevated doses of selenium result in toxicity; therefore, encapsulating selenite into NPs (SeNPs) can significantly lower the toxicity, making them promising for clinical application [135, 136]. Several studies have investigated the anti-atherosclerotic activities of SeNPs in ApoE^−/−^ mice on HFD and demonstrated their successful inhibition of hyperlipidemia and reduced inflammation of blood vessels, as well as strong antioxidant capacity [135, 137].Overall, the use of monoclonal antibodies, gene therapy and drug loaded NPs have proven to be successful in the reduction of LDL receptor expression and consequently plasma cholesterol levels, however, their direct effects on the progression of atherothrombosis has yet to be determined.

#### Regulating cholesterol metabolism in macrophages

By incorporating an agonist to cholesterol-trafficking nuclear liver-X receptors, and thereby regulating cholesterol efflux, Iverson et al. demonstrated less macrophage retention and less cholesterol accumulation in the intimal levels of injured carotid arteries in Sprague–Dawley rats on a high-fat diet (HFD) [105]. Based on the hypothesis that regression of an atherosclerotic plaque can be achieved via reducing the uptake of LDL by macrophages and via stimulating cholesterol efflux, He et al. generated mannose-functionalized dendrimer NPs to deliver the liver-X-receptor ligand and the siRNA against scavenger receptor-A to lesions [138]. This approach inhibited the influx of cholesterol and promoted the efflux of cholesterol, resulting in 20% regression of atherosclerotic lesions in vivo, offering a novel strategy to halt plaque progression by reversing the generation of foam cells [138].

### Smooth muscle cells

Under normal conditions, vascular SMCs with low-rates of proliferation and apoptosis are responsible for sustaining vessel tone, providing structural support, and regulating vessel homeostasis and development. However, during atherosclerosis, vascular SMCs can also acquire the properties of macrophages and contribute to the formation of foam cells [21, 37]. Therefore, another approach to controlling the progression of atherosclerosis is through inhibition of SMC proliferation and limiting their uptake of oxLDL. To do this, some groups have looked at using anti-cancer drugs because they possess an anti-proliferative function; however, some of these drugs may induce neutropenia and lymphopenia. The encapsulation of these drugs into NPs has been shown to provide effective therapy while avoiding side effects. Meneghini et al. loaded Docetaxel, is an anti-microtubule agent used to inhibit cell proliferation, into cholesterol-rich nanoemulsions; and demonstrated an 80% reduction in the atherosclerosis lesion area in the aorta of rabbits on HFD post-therapy [139]. These cholesterol-rich nanoemulsions mimic LDL and enter the cells via LDL-receptor–mediated endocytosis [140]. The rabbit model also showed that this therapy resulted in decreased macrophages and vascular SMCs in the lesions [139].

The use of SMCs to inhibit atherosclerotic progression may require more in-depth studies to be performed because of their involvement in diffused intima thickening in human plaques, a trait that is absent in most animal models [141]. In addition, contradictory results have been observed in preclinical models and clinical trials, where an IL-1β-neutralizing antibody resulted in the rupture of fibroatheromas in murine models [142] while the clinical trial CANTOS showed beneficial results in reducing CVD events [143]. Therefore, there may be a need to have a better understanding of SMCs, in particular to investigate species differences, so that research groups can develop better therapeutics with better prospects of clinical translation.

### Thrombosis

Most acute clinical events are caused by thrombosis over an underlying disrupted plaque [43]. Thrombosis can evoke numerous serious events such as acute coronary syndromes, ischemic stroke, and pulmonary embolism [144]. The pharmacological therapy for these events uses thrombolytic drugs, which activate plasminogen, dissolve fibrin, and break down blood clots [145]. Furthermore, anti-platelet and anti-coagulant drugs are used to prevent or minimize thrombotic events. Anti-platelet drugs work by preventing platelet activation, adhesion, and aggregation. Anti-coagulants diminish thrombin's activity and hinder the activation of a coagulation cascade. However, most of these drugs need to be intravenously injected at high doses, have short half-lives in systematic circulation, and lead to crucial side effects such as bleeding. Therefore, by targeting the biomarkers of thrombosis for site-specific delivery, we may be able to provide low systematic doses, allowing for the enrichment of drugs and increased potency at the site of disease. Several research groups have also created a range of NPs, including hollow nanogels, magnetic particles, and liposomes, for better thrombolysis (Table 4) [144].

**Table 4:** Antibodies and NPs targeting thrombus

Targeting approach	Delivered drugs	Models	Effect	Refs.
Monoclonal antibody targeting the enzymatic pocket of FXIIa	3F7	ApoE^−/−^ on HFD (mice); Tandem stenosis model mimicking vulnerable, rupture-prone plaque (mice)	Decreased stable atherosclerotic plaque burden. Achieved plaque stabilization	[176]
Perfluorocarbon NPs coated on stents	Thrombin inhibitor (PPACK)	In vitro flow model of stent thrombosis	Suppressed growth of thrombi in both static and dynamic models of stent thrombosis	[144]
Fe_3_O_4_ nanorods	tPA	*Ex-vivo* thrombi (rats)	Achieved lysis efficiency of 70%	[148]
Fucoidan-functionalized NPs	tPA	Venous thrombosis (mice)	Significant reduction in thrombus density	[147]
scFv against activated GPIIb/IIIa	scuPA	Acute arterial thrombosis in C57Bl6 and plg^−/−^ (mice)	Displayed successful thrombolysis of blood clots	[160]
	CD39	Acute arterial thrombosis (mice)	Inhibited aggregation of platelets and prevented vessel occlusion	[164]
	CD39	Cardiac I/R (mice)	Preserved cardiac function and significantly reduced infarct size; less cardiac deformation observed using strain analysis	[165]
	TAP	Cardiac I/R (mice)	Preserved cardiac function and significantly reduced infarct size	[161]
	TAP	Acute arterial and electrolytic venous thrombosis (mice)	Reduced thrombolysis post therapy; prophylaxis resulted in reduced thrombosis	[166]
MB + scFv against activated GPIIb/IIIa	scuPA	Acute arterial thrombosis (mice)	Detected and successful in breakdown of thrombi	[162]
Micelles + scFv against activated GPIIb/IIIa	TM	Laser-induced thrombosis in cremaster arterioles (mice)	Reduced platelet deposition and limited thrombus formation	[168]
Mesoporous silica particle–targeted activated GPIIb/IIIa	uPA	In vitro human PRP in static and flow chambers	High affinity to target activated platelets, concentration-dependent thrombolysis	[167]
scFv against red blood cells	TM	Acute venous thrombosis (mice)	Reduced platelet and fibrin deposition at all locations of vascular damage	[169]
NPs + RGD	Aspirin	Acute thrombosis (rats)	Decreased thrombotic risk, restored endothelial barrier integrity, plaque necrosis, and depleted plaque-macrophage content	[151]
	IQCA	Arterial thread thrombosis (mice)	Inhibited thrombosis	[156]
	Lumbrokinase	Acute arterial thrombosis (rats)	Reduced weight of thrombus	[152]
Platelet membrane–coated nanoparticle (PNPs)	Lumbrokinase	Acute arterial thrombosis (mice)	Reduced thrombus area	[155]

#### NPs for thrombolytic drug delivery

The currently used FDA-approved thrombolytic drug for the treatment of acute ischemic stroke is tissue plasminogen activator (tPA), but its usage is limited due to the short therapeutic time window (< 4.5 h) and risk of hemorrhagic complications [146]. The conversion of plasminogen to plasmin is catalyzed by recombinant tPA, a serine protease. Plasmin promotes thrombolysis by degrading the fibrin network of the thrombus [147]. Hu et al. constructed biocompatible tPA-loaded magnetic NPs that could be guided to a blood clot and their drug release triggered locally by an external magnetic field [148]. These magnetic NPs increased thrombolytic efficiency in vivo and lowered the bleeding risk in a rodent study [148]. The magnetic field assists the NPs in accumulating at the location of the thrombus, as well as in stimulating the drug release at the site, resulting in clot lysis efficiency of 70% as compared to 30% with free tPA on its own [148]. P-selectin is an activation-specific biomarker of platelets because it is not expressed by resting platelets in the circulation but is highly expressed upon platelet activation in the thrombus. Using fucoidan, a polysaccharide with a high affinity for P-selectin, Juenet et al. created targeted NPs loaded with tPA and observed a significant reduction in thrombus formation in a murine model of thrombosis [147]. However, P-selectin is also expressed by inflamed endothelial cells; therefore, it is not specific to platelets only. This may result in tPA being directed to endothelial cells and could result in unwanted off-target effects and bleeding problems.

The gold standard anti-thrombotic drug that has been studied in large patient populations and proven to provide a significant reduction in the risk of MI is aspirin [149, 150]. However, some patients are non-responders or have resistance to aspirin where their platelet functions cannot be dampened, thereby impeding their treatment for CVDs [151]. Furthermore, aspirin has also been associated with increased risks of gastrointestinal bleeding and hemorrhagic stroke [149]. To overcome this, Jin et al. generated NPs by conjugating a tetrapeptide, Arg-Gly-Asp-Val (RGDV), with aspirin and demonstrated inhibition of thrombosis in vivo [151]. The effective dose of their site-specific aspirin-targeting NPs was 16,700-fold lower than that of free aspirin [151]. Targeting strategies and NPs encapsulation of aspirin or tPA may allow these drugs to overcome drawbacks, such as short retention time, drug resistance and other side effects.

#### RGD peptides

The Arg-Gly-Asp (RGD) tripeptide sequence is used in many drug-delivery systems to target the proteins within the extracellular matrix for cancer, as well as platelets for anti-thrombotic treatment. Several groups have attempted to use RGD for GPIIb/IIIa targeting in thrombosis, however it is important to note that RGD binds to both the non-activated and activated conformation of this integrin.

Using a cyclic RGD peptide (c-RGD) with better in vivo stability, Liao et al. created targeted chitosan NPs that were coated with lumbrokinase to dissolve fibrin-rich thrombi in Sprague–Dawley rats [152]. This anti-thrombotic drug stimulate adenylate cyclase, increase cAMP level, thereby limiting the increase in intracellular Ca^2+^, and suppressing the expression of GPIIb/IIIa and P-selectin [153]. Using the same drug, Wang et al. fused platelet membranes with PLGA to generate a platelet membrane–coated nanoparticle (PNP) as a carrier [154, 155]. These PNPs were generated from platelet-rich plasma and their membrane proteins allowed them to target thrombosis without modification [155]. The study demonstrated these drug-loaded PNPs showed significant thrombolytic efficacy at a low dose in vivo, with minimal hemorrhagic complications [155].

Wu et al. conjugated a small molecule, 3S-1,2,3,4-tetrathydroisoquinoline-3- carboxylic acid (IQCA), to the RGDV motif to target and inhibit P-selectin and GPIIb/IIIa [156]. This compound was reported to successfully inhibit the activation and aggregation of platelets during thrombosis in murine and rodent models [156]. While the RGD sequence has been used in many studies to target GPIIb/IIIa on platelets, this motif is the principal integrin-binding domain present on fibronectin and vitronectin, as well as being present on collagen, laminins, members of the immunoglobulin superfamily, and plasma proteins [151]. Therefore, Li et al. conjugated cRGD peptides onto PLGA NPs to target the α_v_β_3_ integrin, which is expressed on endothelium during neovascularization [157]. These dexamethasone-loaded NPs were shown to bind to damaged human umbilical vein endothelial cells (HUVECs) and a therapeutic effect of better cell viability was observed in vitro [157]. Therefore RGD has been utilized in numerous studies for targeted drug delivery across the field of cancer, inflammation and thrombosis. Further in vivo experiments may be needed to assess its efficacy and specificity to investigate cross-reactivity and off-target effects. Furthermore, clinically used antiplatelet drugs, such as Tirofiban and Eptifibatide, are mimetics of RGD. They have been shown to bind to highly expressed GPIIb/IIIa receptors on platelets, regardless of their conformation states, which in turn result in fatal bleeding complications [158]. GPIIb/IIa on the surface of platelet is inactive in its default normal state; when platelets are activated, however, the complex changes conformation to a high-affinity state for binding to the plasma protein fibrinogen, which promotes thrombosis. Thus, activated GPIIb/IIa is an ideal therapeutic target for inhibiting thrombosis and preventing atherothrombotic events [158].

#### Single-chain variable fragments against activated GPIIb/IIIa receptors

**Fig. 6:**
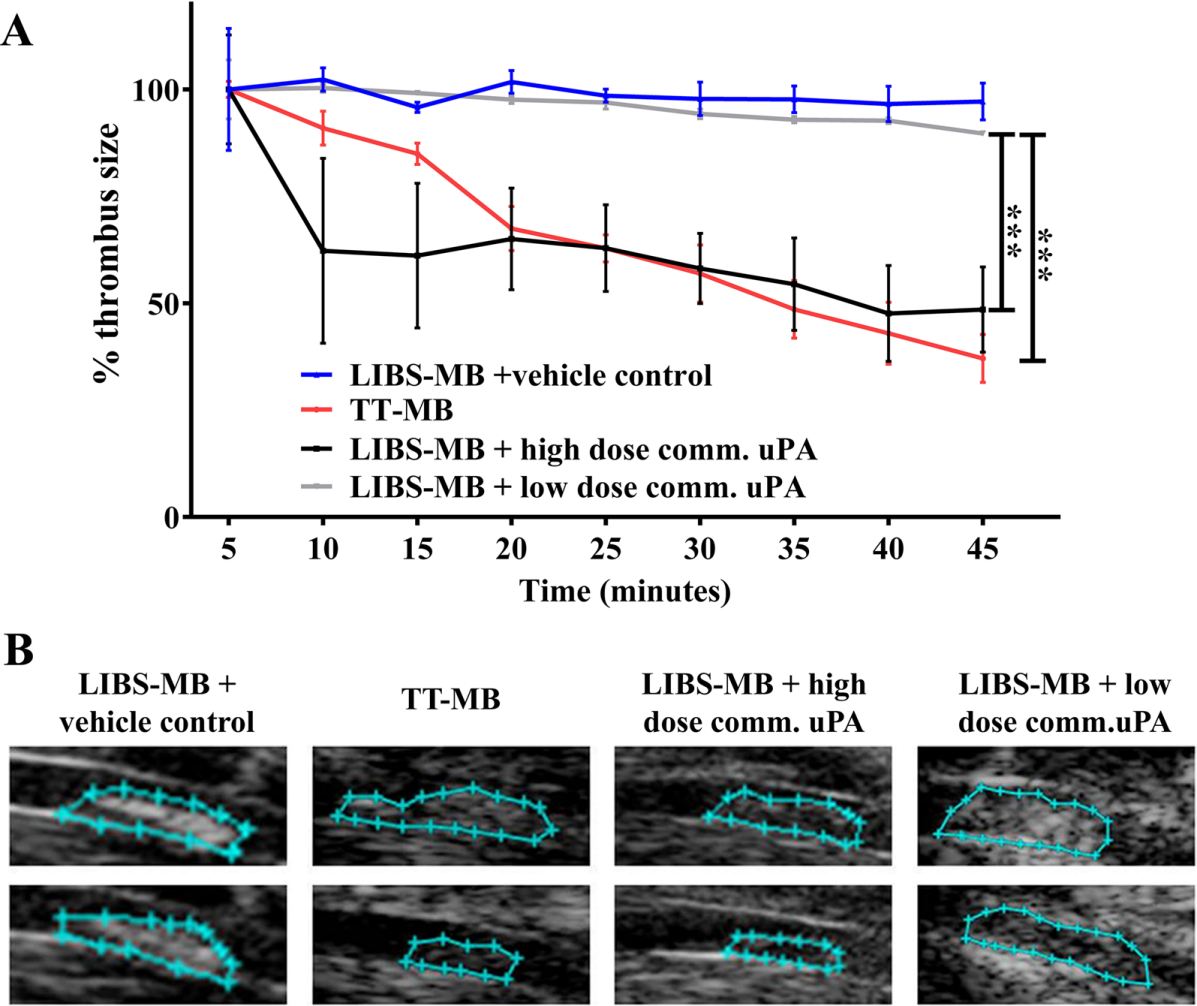
Proof of thrombus theranostic: Monitoring of thrombolysis via molecular ultrasound imaging showed a reduction of thrombus size post administration of TT-MB. **A** A reduction of thrombus size was observed for animals administered with LIBS-MB and high dose of commercial uPA at 500 U/g BW (black line) as compared to LIBS-MB and saline (blue line) as vehicle control. A reduction of thrombus size was also observed with TT-MB (red line) as compared to LIBS-MB and low dose of commercial uPA at 75 U/g BW (light grey line). **B** Representative images of baseline versus 45 minutes post-treatment. Baseline area was set to 100% and areas were calculated every 5 min for 45 min. Thrombus size was traced and calculated using VisualSonics software. Treatment groups were compared by use of repeated measures ANOVA over time with Bonferroni post tests at each time point (Mean % ± SEM, (**p < 0.01, ***p < 0.001, n ≥ 3 each) [161]

To overcome the above issues regarding off-target effects and bleeding complications, recombinant antibody technologies are employed in medical diagnostic and therapeutic applications. Of these, scFvs are one of the most popular choices because they can be genetically modified and are easily produced using different expression systems [159]. The Peter group has developed an scFv that specifically binds to and blocks the fibrinogen pocket of the activated GPIIb/IIIa receptor [158]. Using a fusion of this scFv to a single-chain urokinase plasminogen activator (Targ-scuPA) in a mouse model of thrombosis, Wang et al. demonstrated the successful thrombolysis of blood clots in real time on ultrasound imaging in vivo [160]. This scFv and scuPA was also conjugated onto MBs in an in vivo theranostic approach for simultaneous diagnosis and treatment (Fig. 6) [161]. In this study, the authors demonstrated that these dual-functional, targeted theranostic MBs successfully enabled visualization of thrombosis in ultrasound imaging, as well as dissolving the clots and providing direct monitoring of thrombolysis in real time [162]. The use of scuPA, a fibrinolytic drug may be an ideal therapeutic candidate for the breakdown of an established clot and has shown great potential to be used in a theranostic strategy.

Hohmann et al. fused the ectonucleoside triphosphate diphosphohydrolase-1 (CD39) to the activated GPIIb/IIIa–specific scFv (Targ-CD39) and demonstrated anti-platelet and anti-inflammatory effects across several murine models [163–165]. CD39 is responsible for hydrolyzing pro-inflammatory ATP and pro-thrombotic ADP to anti-inflammatory AMP; however, the systemic administration of CD39 contributes to a severe bleeding complication [165]. By using Targ-CD39 to target only the activated platelets at the site of thrombosis, this construct was effective in the inhibition of platelet aggregation in vitro, as well as the prevention of vessel occlusion in two acute thrombosis murine models in the mesenteric and carotid arteries [164]. To achieve efficiency in vivo, the non-Targ-CD39 (active CD39 that was genetically fused to a non-binding scfv as a control) required a dose 10 times higher and resulted in prolonged bleeding [164]. The small dose of Targ-CD39 needed in vivo did not result in systemic overload or prolongation of bleeding time [164]. Ziegler et al. also demonstrated thatTarg-CD39 successfully preserved cardiac function in the treated mice in an MI I/R murine model [165]. In this study, the authors also observed significant cardiac deformation via radial and longitudinal strain and strain rate in vivo, as well as increased inflammatory response in the control animals [165]. The use of soluble CD39 have been previously associated with high rate of bleeding side effect. Fusion of CD39 with GPIIb/IIIa-targeted scFv allowed Targ-CD39 to achieve the delicate balance between fibrinolytic therapy and bleeding side effects. Future in vivo experiments should investigate CD39’s dual anti-thrombotic and anti-inflammatory potency for therapy of atherothrombosis.

**Fig. 7:**
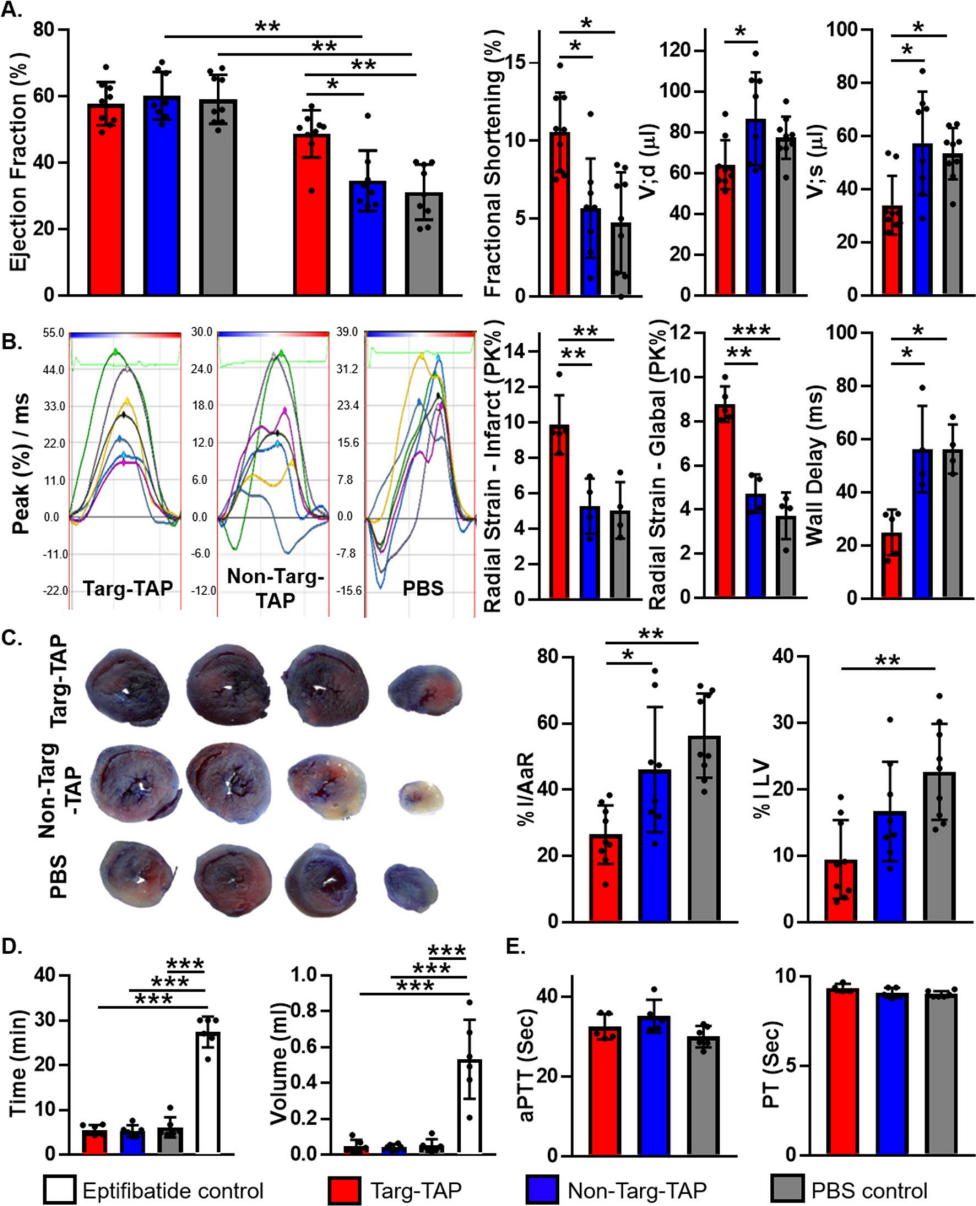
Targ-TAP preserves myocardial function and reduces infarct size after I/R. **A** 4-week post-I/R, EF is preserved in Targ-TAP-treated mice as compared to baseline, Non-Targ-TAP or control-treated mice. Targ-TAP also improved FS and prevented V;d and V;s LV dilatation. **B** Representative radial strain curves. Colored lines represent the six myocardial regions, black lines represent the average (global) strain. Control mice exhibit a marked decrease in radial strain, both in the infarcted area and globally compared to Targ-TAP. Control mice show significant increases in time for maximum opposite-wall delay as compared to Targ-TAP. **C** Representative images of Evans Blue/TTC stained hearts 4 weeks post-I/R. Targ-TAP-treated hearts show a reduced I/AaR ratio and infarct size compared to controls. **D** No difference is observed for bleeding time and blood loss in mice injected with Targ-TAP, compared to control; both are significant increased in mice injected with Eptifibatide. **E** Targ-TAP did not show a difference in aPTT or PT compared to controls. After normality was confirmed (Anderson–Darling and Shapiro–Wilk tests), one-way or two-way ANOVA with Tukey’s post-hoc test were applied [161]

Another scFv–drug fusion construct from this group uses the tick anti-coagulant peptide (TAP). Hanjaya-Putra et al. demonstrated that the dual-function anti-platelet/anti-coagulant drug (Targ-TAP) displayed excellent anti-thrombotic capacity across several murine models without significant bleeding influence [166]. Using an MI I/R murine model, Bienvenu et al. showed that Targ-TAP treated mice had preserved heart function on echocardiography and significantly reduced infarct size on histology, as compared to the control group (Fig. 7) [161]. Being a Factor Xa inhibitor, TAP prevents further activation of the coagulation cascade; therefore, it is an ideal candidate for thromboprophylaxis. The employment of Targ-TAP in a prophylactic approach allows up to four hours of protection from thrombosis [166], indicating that further developments of such targeted-drug should also focus on extending their circulating half-life. The ability to provide longer protection will result in less administrations, and therefore cost, which will be an important step for future clinical translation.

In addition to antibody–drug fusion proteins, research groups have conjugated scFvs onto NPs for targeted delivery [167, 168]. Gunawan et al. designed a layered assembly of mesoporous silica conjugated with a thrombin-cleavable sequence and scFv to generate a multifunctional polymer nanocarrier system for delivery of commercially available urokinase plasminogen activator [167]. These thrombin-cleavable polymer capsules allowed them to control drug release in the microenvironment of the thrombus [167]. The in vitro results showed that the particles had a high affinity to targeting activated platelets and displayed concentration-dependent thrombolysis [167]. Using elastin-based protein polymers, Kim et al. developed targeted multifunctional protein micelles functionalized with both human thrombomodulin fragments and the scFv specific to activated GPIIb/IIIa receptors [168]. In vivo assays demonstrated that the targeted micelles displayed site-specific binding and significantly reduced platelet deposition and thrombus formation [168]. Zaitsev et al. devised another delivery strategy for thrombomodulin by targeting the membranes of circulating red blood cells and demonstrated significant reductions in platelet and fibrin deposition at the site of vascular damage in vivo [169]. Overall, both direct targeting and NP platforms provided therapeutic effects, however the NP delivery platforms may be more flexible, enabling the incorporation of multiple drugs, which will provide more opportunities towards much needed therapeutic approaches in CVD.

## Future directions and limitations

Targeted therapies and the use of NPs as emerging technologies have attracted major interest in many areas of medicine, including cancers, CVDs, and neurodegenerative disorders. Targeted therapies using peptides, antibodies, and related recombinant proteins have many advantages because of their potential for clinical translation. Most of the current applications require intravenous injection; however, as these strategies develop, there may be formulations that are easier to use subcutaneously or by inhalation.

NPs are used to protect drugs from degradation, carry therapeutic agents to target sites, and facilitate controlled release [48]. In most studies, NPs have been employed in drug delivery for their properties of passive accumulation and phagocytosis by macrophages. The small size of NPs helps them to penetrate capillaries and pass through cell membranes. The flexibility of creating these NPs using a wide range of biomaterials which are biocompatible and biodegradable allows them to be created to provide slow and long-lasting release of drugs. The most established type of particle is nanoliposomes, for several reasons: they are easy to prepare, potential to be modified, have excellent biocompatibility, and have the ability to transport both hydrophilic and lipophilic drugs [170]. Another advantage of nanoliposomes is their ability to enhance gene therapy, in carrying and transferring nucleic acids across the cell membrane. As compared to other gene-therapy vectors such as viral vectors, nanoliposomes exhibit high transgenic efficiency without toxicity [83]. In fact, the fast-tracking of these nanoliposomes is evident in the recent approvals of COVID-19 mRNA-based vaccines [51–55]. Nevertheless, these non-targeted NPs have been criticized for their off-target effects and unpredictable side effects. Therefore, some groups have employed ligands to directly target the NPs to specific biomarkers for active targeting, whereas others have used stimuli-responsive NPs that require external stimuli (ultrasound, plasmonic or magnetic responsiveness) for drug delivery [48, 63, 162, 171, 172].

Despite all the benefits and the preclinical research conducted in these fields, we must be mindful that there are some limitations to the use of NPs in CVDs. Most NPs are produced in small amounts for preclinical studies and may suffer batch-to-batch variations or face difficulties in upscaling [173]. While some NPs are in clinical use [51–55, 174], no more than 20 clinical trials have been registered at ClinicalTrials.gov (search terms “nanoparticle” and “cardiovascular”); therefore, the FDA and European Medicines Agency have yet to approve any cardiovascular nanomedicine products. Effective translation will require the particles to overcome several challenges, including scale-up, drug-loading efficiency, drug-release profile, and stability [175]. The complexity of CVDs requires disease-driven creation of NPs instead of formulation-driven generation. Overall, the applications of cardiovascular nanomedicine for patients are still in their early stages and a considerable amount of effort will be required to achieve clinical breakthrough.

## Conclusion

The cost burden for the management of CVDs is huge. Despite clinical attempts to mitigate risk factors, the progression of atherosclerosis and atherothrombosis is still the cause in one of four deaths. To overcome this, more effective methods are required to improve drug delivery, achieve high therapeutic efficacy, and reduce side effects. Advances in the biotechnology for targeted therapy using peptides and recombinant proteins, as well as nanotechnology for therapeutic-loaded nanomedicine platforms, are attracting major interest. In particular, clinical approvals for cancer therapies and the high-profile clinical approvals of multiple mRNA vaccines for COVID-19 have set the stage for these therapies in CVDs. With further advances in targeted therapies and bio-/nano-technologies developed to combat atherosclerosis and atherothrombosis, the clinical translation of these methods will benefit patients across a broad spectrum of CVDs.

## Data Availability

Not applicable.

## Abbreviations

AAA: Abdominal aortic aneurysm
ABC: ATP-binding cassette transporter
ADP: Adenosine diphosphate
AM: Amphiphilic macromolecule
AMP: Adenosine monophosphate
Apo: Apolipoprotein
ATP: Adenosine triphosphate
C6-KH: Lysine- and histidine- oligopeptide-modified pBAE
CCL: Chemokine (C–C motif) ligand
c-RGD: Cyclic RGD peptide
CVD: Cardiovascular disease
CXCL: Chemokine (C–X–C motif) ligand
DARPin: Designed ankyrin repeat protein
DOPE: Dioleoyl-phosphatidyl-ethanol-amine
F: Factor
FDA: Food and Drug Administration
GP: Glycoprotein
HA: Hyaluronic acid
HAEC: Human aortic endothelial cell
HFD: High-fat diet
HUVEC: Human umbilical vein endothelial cell
ICAM: Intercellular cell adhesion molecule
IL: Interleukin
IQCA: 3S-1,2,3,4-tetrathydroisoquinoline-3-carboxylic acid
JNK: Jun N-terminal kinase
LDL: Low-density lipoprotein
LDLR: Low-density lipoprotein receptor
LOX-1: Lectin-like oxidized low-density lipoprotein receptor
mAb: Monoclonal antibody
Mac-1: αMß2, CD11b/CD18, complement receptor 3
MB: Microbubble
MCP-1: Monocyte chemotactic protein-1
MI: Myocardial infarction
miR: MicroRNA
MMP: Matrix metalloprotein
mRNA: MessengerRNA
MSR-1: Scavenger receptor A1
NP: Nanoparticle
OxLDL: Oxidized low-density lipoprotein
PAR: Protease-activated receptor
pBAE: Poly-beta-amino ester
PCSK9: Proprotein convertase subtilisin/kexin type 9
PECAM-1: Platelet endothelial cell adhesion molecule-1
PLGA: Poly(lactic-co-glycolic acid)
PNP: Platelet membrane–coated nanoparticle
PPARγ: Peroxisome proliferator–activated receptor gamma
rHDL: Reconstituted high-density lipoprotein
RhoA: Ras homolog gene family, member A
ROS: Reactive oxygen species
SARS-CoV-2: Severe acute respiratory syndrome coronavirus 2
SCAP: SREBP cleavage-activating protein
scFv: Single-chain variable fragment
scuPA: Single-chain urokinase plasminogen activator
SeNP: Selenium nanoparticle
sLeX: Sialyl LewisX
SM22α: Smooth muscle protein 22α
SMC: Smooth muscle cell
SR: Scavenger receptor
SRE: Sterol-regulatory element
SREBP: Sterol regulatory element–binding protein
TF: Tissue factor
TNF-α: Tumor necrosis factor-α
tPA: Tissue plasminogen activator
VCAM: Vascular cell adhesion molecule
vWF: von Willebrand factor
